# Uncertainty management in regulatory and health technology assessment decision-making on drugs: guidance of the HTAi-DIA Working Group

**DOI:** 10.1017/S0266462323000375

**Published:** 2023-06-16

**Authors:** Milou Amber Hogervorst, Rick Vreman, Inkatuuli Heikkinen, Indranil Bagchi, Inaki Gutierrez-Ibarluzea, Bettina Ryll, Hans-Georg Eichler, Elena Petelos, Sean Tunis, Claudine Sapede, Wim Goettsch, Rosanne Janssens, Isabelle Huys, Liese Barbier, Deirdre DeJean, Valentina Strammiello, Dimitra Lingri, Melinda Goodall, Magdalini Papadaki, Massoud Toussi, Despina Voulgaraki, Ania Mitan, Wija Oortwijn

**Affiliations:** 1 Division of Pharmacoepidemiology and Clinical Pharmacology, Utrecht Institute for Pharmaceutical Sciences (UIPS), Utrecht University, Utrecht, The Netherlands; 2 National Health Care Institute (ZIN), Diemen, The Netherlands; 3 Global Regulatory Policy, MSD, Copenhagen, Denmark; 4 Global Pricing & Market Access, GSK, Philadelphia, PA, USA; 5 Basque Foundation for Health Innovation and Research (BIOEF), Barakaldo, Spain; 6 Basque Office for Health Technology Assessment, Osteba, Barakaldo, Spain; 7 Melanoma Patient Network Europe, Uppsala, Sweden; 8 Association of Austrian Social Insurance Bodies, Vienna, Austria; 9 Department of Health Services Research, CAPHRI Care and Public Health Research Institute, Maastricht University, Maastricht, The Netherlands; 10 Health and Society Lab, Faculty of Medicine, University of Crete, Heraklion, Greece; 11 Center for Evaluation of Value and Risk in Health, Tufts Medical Center, Boston, MA, USA; 12 Global Public Affairs, Novartis, Basel, Switzerland; 13 Department of Pharmaceutical and Pharmacological Sciences, KU Leuven, Leuven, Belgium; 14 Canadian Agency for Drugs and Technologies in Health, Ottawa, ON, Canada; 15 European Patients’ Forum, Brussels, Belgium; 16 European Healthcare Fraud and Corruption Network – EHFCN, Brussels, Belgium; 17 Aristotle University of Thessaloniki, Thessaloniki, Greece; 18 Regulatory Affairs International, MSD (UK) Limited, London, UK; 19 Real World Evidence Solutions, IQVIA, Courbevoie, France; 20 Medtronic Ltd, Watford, UK; 21 Maastricht University, Maastricht, The Netherlands; 22 DIA, Basel, Switzerland; 23 Department for Health Evidence, Radboud Institute for Health Sciences, Radboud University Medical Centre, Nijmegen, The Netherlands; 24 Health Technology Assessment International (HTAi), Edmonton, AB, Canada

**Keywords:** uncertainty, drugs, health technology assessment, regulatory decision making, stakeholder deliberation

## Abstract

**Objectives:**

Uncertainty is a fundamental component of decision making regarding access to and pricing and reimbursement of drugs. The context-specific interpretation and mitigation of uncertainty remain major challenges for decision makers. Following the 2021 HTAi Global Policy Forum, a cross-sectoral, interdisciplinary HTAi-DIA Working Group (WG) was initiated to develop guidance to support stakeholder deliberation on the systematic identification and mitigation of uncertainties in the regulatory-HTA interface.

**Methods:**

Six online discussions among WG members (Dec 2021–Sep 2022) who examined the output of a scoping review, two literature-based case studies and a survey; application of the initial guidance to a real-world case study; and two international conference panel discussions.

**Results:**

The WG identified key concepts, clustered into twelve building blocks that were collectively perceived to define uncertainty: “unavailable,” “inaccurate,” “conflicting,” “not understandable,” “random variation,” “information,” “prediction,” “impact,” “risk,” “relevance,” “context,” and “judgment.” These were converted into a checklist to explain and define whether any issue constitutes a decision-relevant uncertainty. A taxonomy of domains in which uncertainty may exist within the regulatory-HTA interface was developed to facilitate categorization. The real-world case study was used to demonstrate how the guidance may facilitate deliberation between stakeholders and where additional guidance development may be needed.

**Conclusions:**

The systematic approach taken for the identification of uncertainties in this guidance has the potential to facilitate understanding of uncertainty and its management across different stakeholders involved in drug development and evaluation. This can improve consistency and transparency throughout decision processes. To further support uncertainty management, linkage to suitable mitigation strategies is necessary.

## Introduction

Regulators perform benefit-risk assessments, thereby safeguarding that only the treatments for which the benefits outweigh the risks are licensed and which allows them to be provided to patients. On the other hand, payers use health technology assessment (HTA) for pricing and reimbursement decisions. HTA is a multidisciplinary process aimed at determining the value of an intervention, in service of promoting an equitable, efficient, and high-quality healthcare system (1;2). By the very nature and complexity of health technologies, as well as methodological limitations associated with their assessments, uncertainty exists to some degree in both regulatory and HTA-informed decision making.

Knight defined uncertainty as “the lack of knowledge about the probabilities of the future state of events” (3). Recent definitions also include the element of “lacking complete knowledge or understanding of a given situation” (4). In the context of regulatory and HTA-informed decision making uncertainty may translate into unpredictability of outcomes, that is, lacking clarity from the evidence or data available to evaluate the (relative) safety, efficacy, quality, (cost-)effectiveness, and/or budget impact of a medical product for a given patient population. The relevance of uncertainty in specific decision making activities is highly context-specific, depending for example on established social values or practices in a specific country, at institutional or individual level, or in a specific disease area. Some shared standards or values may be embedded in rules and legislation (5), while others may be more implicit.

Key challenges and opportunities regarding uncertainty in HTA have been discussed during the 2021 Health Technology Assessment International (HTAi) Global Policy Forum (GPF) (6;7). Building on the recommendations of the 2021 HTAi GPF, a Working Group (WG) was initiated by HTAi and the Drug Information Association (DIA) to provide guidance on management of uncertainty in the regulatory-HTA interface across the drug’s lifecycle (8;9).

Uncertainty management refers to carefully balancing the benefits of reducing uncertainty with the efforts of doing so and the opportunity costs of the alternatives (2). The added value of reducing uncertainty has many dimensions, for example, improved knowledge on treatment efficacy or safety, a reduced possibility for decision errors, a better understanding of the relative effectiveness or associated costs reducing the risk of healthcare displacement or efficiency in the design and implementation of solutions (2;6;10–13). The costs associated with uncertainty reduction in terms of additional evidence generation can be tackled to some extent through value of information (VOI) analyses (14). This trade-off typically includes the time spent on the generation of new evidence and the size of the involved population but to a lesser extent the ethical considerations that may affect the trade-off (15;16). The implications of accepting uncertainties (i.e., being risk tolerant) and their opportunity costs are often more difficult to quantify (2). In order to have effective stakeholder deliberation on these trade-offs, a clear understanding of what constitutes uncertainty is required. Regulators and HTA organizations might benefit from a tool that supports the identification and classification of uncertainty to facilitate deliberation and develop mutually accepted mitigation strategies. Additionally, the role of and interaction with other stakeholders, for example, the patients’ voice is becoming increasingly important when it comes to the willingness of accepting uncertainties, as is elicited through patient preference studies, patient-reported outcomes, or patient consultations. This also relates to the differences among stakeholders on facing the risks (less or more risk aversion).

The aim of this paper is to provide guidance on uncertainty management in the context of regulatory and HTA-informed decision making on drugs, by:

1. *Defining* uncertainty: Defining the main contributing factors in relation to what decision makers perceive as uncertainty;
2. *Identifying* uncertainty: Identifying key stakeholder considerations on how to identify relevant uncertainties within different contexts, accounting for various perspectives;
3. *Mapping* uncertainty: Developing a consistent and transparent way to systematically classify uncertainty;
4. *Mitigating* uncertainty: Exploring the use of the guidance and the development of strategies to prevent or reduce uncertainty through a real-world case study.

## Methods: development of the guidance

### Formation of the Working Group

The authors WO and IH have been leading the HTAi-DIA WG and invited participants based on (research) expertise related to uncertainty, geography to represent a global group of participants, and work environments, including the regulatory, HTA, payer, developer, and patient perspective. In total, 29 experts were invited of which one did not accept due to capacity reasons, and another expert resigned after the first meeting, resulting in a WG of 27 experts. Six WG calls have taken place between Dec 2021 and Sept 2022, including one scoping meeting and five deep dives. During the scoping meeting the background, the objectives, outputs, and process of the WG were discussed. During that meeting, it was also decided to focus on handling uncertainties related to drugs in the regulatory-HTA interface, since regulation for assessment of devices and vaccines differs significantly. The topics of the deep dive meetings concerned (i) existing frameworks and definitions for uncertainty in the regulatory-HTA interface, (ii) conducting a survey on uncertainties in two literature-based case studies, (iii) defining uncertainty through codifying key factors of uncertainty into building blocks and taxonomizing literature and survey results into an uncertainty map, (iv) converting building blocks into a checklist relevant for identification of uncertainty in the regulatory-HTA interface, and (v) exploring mitigation strategies for uncertainty or the associated risks.

### Scoping Review

For the development of the guidance and in preparation of the WG meetings, a scoping review was performed to identify examples of definitions and frameworks for regulatory and HTA-related uncertainty considerations. Literature was gathered through a PubMed search in article titles using the terms “uncertain” or “risk” combined with either “HTA” or “regulator,” “authorization,” “European Medicines Agency,” or “Food and Drug Administration.” Additionally, definitions of uncertainty were gathered from dictionaries such as the Cambridge Dictionary and the HTA Glossary (17;18). Finally, articles were selected using the tool from Connected Papers (19). Based on a list of definitions, WG members voted for the preferred definition. This list of definitions served as input for the building blocks that define uncertainty and the uncertainty map that taxonomizes uncertainty.

### Literature-Based Case Studies and WG Survey

Next to the scoping review, two literature-based case studies were selected based on previous research output from the authors (RV and MH) and agreed by all members as being of high informative value, being (i) a tumor-agnostic treatment, and (ii) a treatment for cystic fibrosis. Various uncertainties were extracted from these case studies and surveyed among the WG members (see Supplementary Table 1 for the survey). The aims were to identify various types of uncertainty (the kind of uncertainty or characteristics of the uncertainty, e.g., something unknown, unclear) and domains of uncertainty (where does the uncertainty exist or stem from or what is it exactly that is uncertain, e.g., trial population, the magnitude of effects, budget impact). Together with the scoping literature review results, the aspect discussed with the WG focused on how the uncertainties from the survey may be perceived by different stakeholder groups, assessing whether WG members considered the issues presented an uncertainty and why they may have done so or not. This information was used to develop the uncertainty map (taxonomy), validate the definition building blocks, and translate the building blocks into the checklist that may be used to identify uncertainty in the decision-making context.

### Real-World Case Study and International Conference Panels

Interim results of the WG activities were presented at the DIA Europe conference in Mar 2022. Final results including an exemplifying real-world case study from one of the WG members was presented at the HTAi Annual Meeting in June 2022. The panel discussions and audience feedback were instrumental in drafting the guidance. A real-world case identified by the WG was used from a pharmaceutical developer perspective to test and fine-tune the guidance, as well as to exemplify how the guidance may be used in stakeholder deliberation on developing mitigation strategies for uncertainty and corresponding risks. This real-world case as well as the application of the guidance to this case study are described in the results.

### Content of the Guidance

The guidance consists of four parts, in line with the objectives of the paper. First, the guidance provides *building blocks* of uncertainty in regulatory and HTA decision making, which form the basis for the *definition* of uncertainty. Second, the guidance presents a *checklist* to be used for the *identification* of the relevant uncertainties as part of decision making or to help inform regarding potential uncertainties that may be relevant to one or more stakeholders. Third, an *uncertainty map* is offered presenting the domains in which relevant uncertainties for regulators or HTA organizations may exist. The checklist may be used in combination with this map in order to identify the relevant uncertainties for each of these domains in a consistent and transparent manner. Fourth, the results of applying the guidance to a real-world case study are included.

### Target Audience and Application

The guidance is envisioned to be used throughout the drug lifecycle, aiming to facilitate deliberation between stakeholders, predominantly focusing on the uncertainty trade-offs in regulatory and HTA decision making (20). Pharmaceutical developers may leverage the guidance to inform internal decision making in relation to plan for regulatory and HTA applications, by discussing evidence expectations of decision makers, potential anticipated data gaps as well as for the development of potential mitigation strategies. This can take place both prior to the finalization of the pivotal trial program as well as regarding post-launch evidence generation. Regulatory authorities and HTA organizations may use the guidance to anticipate and assess impact of different factors leading to uncertainty and codevelop mitigation strategies with the developer, as well as discuss or communicate strategies and considerations with each other. During and after regulatory approval and HTA, the guidance may be used to communicate uncertainties to health professionals and patients and/or to reassess uncertainties based on the established mitigation strategies and considering emerging post-launch data (lifecycle approach). This list of identified stakeholders and applications is not meant to be exhaustive, other stakeholders and/or applications may exist, including for example potential relevance for payers and in the context of preparedness(21). However, these are not explicitly within the scope of this manuscript.

## Defining Uncertainty: The Building Blocks

**Figure 1.**
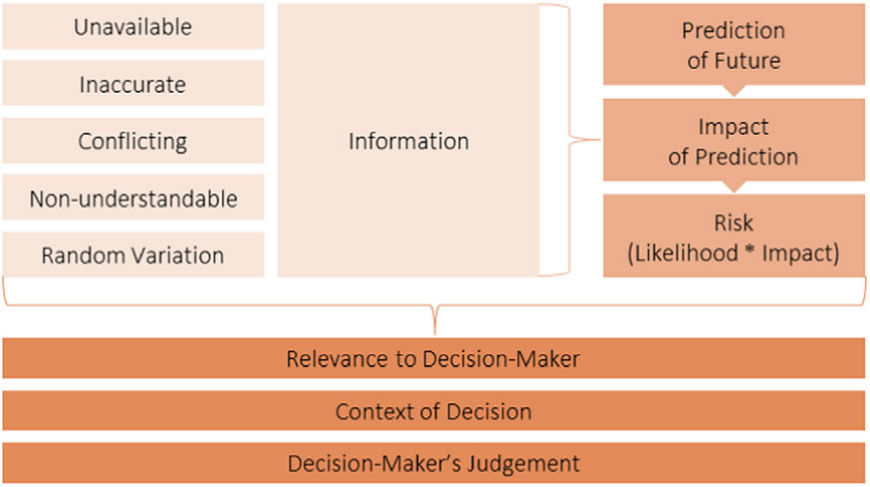
Visualization of the link between the building blocks comprising decision-making uncertainty.

As the WG did not reach consensus on a worded uniform definition of uncertainty in the regulatory-HTA interface, the WG agreed it would be valuable to identify the building blocks that together construct the various perceived aspects of uncertainty. Table 1 provides an overview of the building blocks, synonyms for these and a short description, and Figure 1 visualizes how these building blocks link to each other. These building blocks are based on definitions in literature, dictionaries/glossaries, definitions provided by WG members, and the arguments that WG members provided when considering the literature-based case study examples. Various terms may be used to express these building blocks.

**Table 1.** Building blocks comprising decision-making uncertainty in the regulatory-HTA interface

Building block	Alternative or descriptive wording	Description
Unavailability	Available, existing, accessible, lacking, deficient, a lack of, limitation, insufficient, missing, unknown	Inability to access data or, data nonexistent
Inaccurate	Biased, Imprecise, (not) true, unreliable, definite, indirect, parameter and structural uncertainty	Quality of data or evidence
Conflicting	Inconsistent, clashing, incompatible, opposing, paradoxical, contradictory	Variation in results across information sources
Not understandable	Nontransparent, not concise, inconsistent, incomprehensible, difficult interpretation, confusing, (health) literacy	Information not presented in an understandable format
Random variation	Heterogeneity, stochastic uncertainty, variation, distribution	Inherent, naturally existing differences in data
Information	Knowledge, evidence, measurements, results	The information or data that contains the “flaw”
Prediction of future	Likelihood, could be, chance, unpredictability, in the future, potential	Proxy of reality with future element
Impact of prediction	Negative impact, outcomes	The decision or counterfactual has an outcome or impact
Risk	Reduced confidence, negative outcome, likelihood times impact	Negative nature of the impact of the decision or counterfactual
Relevance to decision maker	Importance	Relevance to the decision in specific context
Context of decision	Given question, specific decision, conclusion, recommendation	Context in which the decision is made
Judgment of decision maker	Sufficient, wrong, undesired, undecided, range	Judgment of decision maker on the uncertainty

The lighter orange blocks in Figure 1 represent the *potential flaws that could exist in information* and the narrowest and more scientific approach of defining uncertainty. *Unavailable, biased, conflicting*, or *incomprehensible* information represent epistemic, parametric, and structural forms of uncertainty. These flaws in knowledge are theoretically preventable, through additional research or different study designs (not considering the practical constraints, e.g., including a thousand additional patients in a trial). *Unavailable* information would also entail information that is inaccessible in certain countries or incomprehensible. *Random variation* that exists in a population is the type of uncertainty that represents the aleatoric or stochastic form of uncertainty. This type of uncertainty is nonreducible and unpreventable, although it may be quantified and visualized (22).

The medium-light orange blocks in Figure 1 represent the *decision making* aspect in uncertainty and includes risk in the equation. Decision making entails predicting how a certain decision (or its alternative) will play out in the future and how likely it is to occur. HTA by definition is the way of presenting policy alternatives and their consequences (1). In other words, what consequences may the decision have or what risks may be associated with the decision. In regulatory decision making, for example, this could include the Type I or Type II errors(2). The risks resulting from an uncertainty or from the decisions made to mitigate the uncertainty are completely different from the uncertainty itself, but nevertheless crucial to the decision.

The darker orange blocks in Figure 1 relate to the decision making *context.* WG members indicated that merely not having certain information may just be a fact, and only becomes relevant if it is related to the decision question to be answered. This relevance is always dependent on the context, for example, who makes the decision, at what stage in the lifecycle, in what country or region, from which perspective an assessment (e.g., regulatory, HTA, health care system or societal) may be undertaken, and so on. Eventually, the decision maker’s judgment on the importance of the lacking information and the potential impacts this may have determine whether it constitutes a relevant uncertainty. Relevance is a key word here as irrelevant uncertainties may have limited or no impact and the possibility to entirely disregard them can be plausible.

The building blocks were developed based on multiple discussions and a survey among WG members. In total 19 different definitions of uncertainty were identified in the literature or provided by the WG members (4;17;18;23–31). See Supplementary Table 2 for an overview of all definitions and voting of WG members for their preferred definition. There was no consensus among WG members regarding the definition. Some of the definitions were considered to be too broad or generic for the regulatory-HTA interface, lacking the necessary contextual relevance to the regulatory and HTA decision-making processes (e.g., “*a lack of precise knowledge*”). Others were too specific or narrow, not being applicable in settings relevant to all potential stakeholders (e.g., “*a deficiency in the information available for a given question, such that the conclusion, decision or recommendation is unknown or not definite*”). The appropriate choice of words led to discussion among WG members and was deemed to be context-dependent. The discussion touched upon the distinction between the origin (what it is that we do not know) or the effect (what the impact of not knowing may be) of uncertainty. This is key to determining whether the uncertainty is important to be resolved or not. Additionally, the aleatoric and epistemic nature of uncertainty were discussed.

## Identifying Uncertainty: Stakeholder Considerations

**Figure 2.**
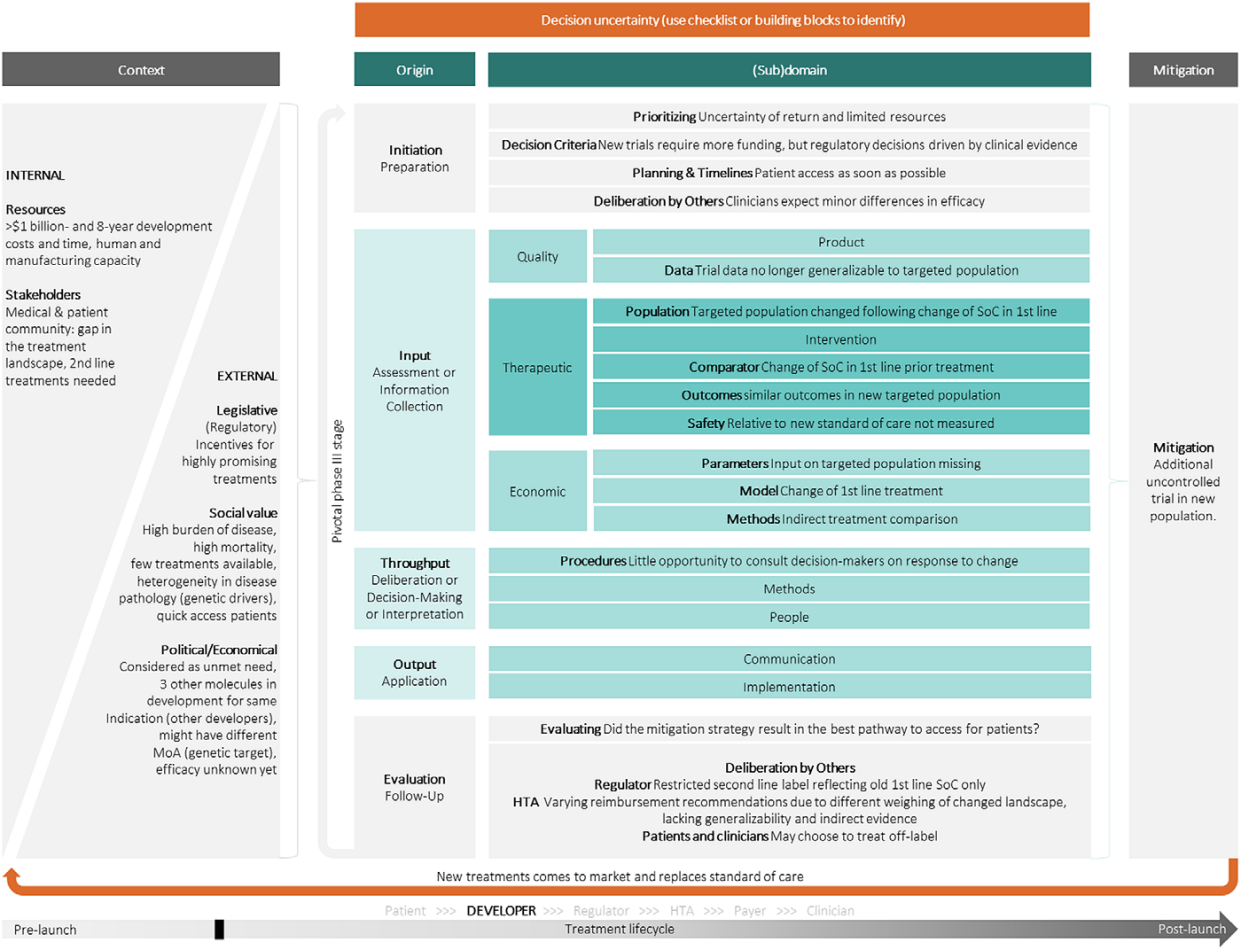
Uncertainty map covering the domains where uncertainty could exist for regulatory and/or HTA-informed decisions.

The WG focused primarily on the context of the regulatory-HTA interface and deliberative processes at different points across the drug lifecycle (20). Not all knowledge gaps (i.e., unavailable, biased, conflicting, incomprehensible, random variation) create an uncertainty that is relevant to (i.e., affects decision) or addressable (i.e., ability to consider) in a given decision. Whether an uncertainty is relevant in that given decision making context may depend on the impact that it has to the decision maker and its stakeholders, as well as the associated risk(s). How the importance of the uncertainty is judged may depend on the nature of uncertainty, that is, whether it is preventable (unavailability, biased, conflicts, or incomprehension) or whether it can be anticipated (possibly also random variation). Therefore, the building blocks were translated into a short checklist to guide the assessment of importance of any potential lack of information, shown in Table 2. The checklist can be used in combination with the map presented in the next section (Figure 2).

**Table 2.** Tool for the identification of uncertainties that may need mitigation

Question to be answered from the decision makers’ perspective:
Is there an uncertainty? Is information *unavailable*? Is information *inaccurate*? Is information *conflicting*? Is information *not-understandable*?
Does the uncertainty impose a *risk (likelihood × negative impact)*? If not mitigated If mitigated
Is the uncertainty *relevant* to you and your *context*? Does it affect your decision?
Can the uncertainty be *anticipated* on? Are you able to know upfront that this uncertainty might or will exist?
Can the uncertainty be *prevented*? Are you able to prevent or reduce the uncertainty once you know it exists?
Based on your *judgment*, should the uncertainty be *mitigated*? Is it, from your perspective, worthwhile to mitigate the uncertainty, based on the balance between the positive and negative implications of mitigating and not/differently mitigating?

## Mapping Uncertainty: Developing a Consistent and Transparent Way to Systematically Map Uncertainties

Through the scoping review and input from the WG, sixty-six papers on uncertainty in the regulatory and HTA field were identified. These were used as input for the uncertainty map. The list of the selected articles is presented in Supplementary Table 3. Based on all the identified uncertainties in the literature and iterative discussions with the WG, the uncertainty map in Figure 2 was constructed.

The uncertainty map is intended as a tool for deliberation between stakeholders about identified uncertainties, mitigation strategies, and thereby provides collective accountability in the drug development, assessment, and approval process. The map can be also a comparator tool, with stakeholders completing it for a given situation, for example, in the context of early scientific advice, to identify areas of convergence and divergence. Internal and external contextual factors influence the scope of dialogues on uncertainties and are usually established “around the table,” that is, in deliberation (32).

The structure of the map is based on the input-throughput-output framework used in the 2021 HTAi GPF (6) and supplemented by an initiation and evaluation step before and after the actual assessment. As described in the article on the outcomes of the 2021 HTAi GPF (6), input refers to the collection of information, evidence, and perspectives with an exploration of the presence and impact of uncertainty. Throughput pertains to the deliberative or decision-making phase with a critical examination of the evidence and weighting the uncertainty and its impact. Lastly, output covers the communication of the decision outcome, the level of uncertainty, its impact, and any actions (such as recommendations for more evidence generation). In each step of the process, several different types of uncertainties may exist. This process is highly contextual, represented by the left gray part in Figure 2. Both internal and external factors should be considered. For each of the uncertainties identified, a mitigation strategy could be considered through deliberation with other stakeholders, represented by the right gray part in Figure 2. Many uncertainties will exist in every individual deliberation cycle, indicated by the orange arrow underneath the map. Each of these uncertainties may be identified using the checklist in Table 2. Supplementary Table 4 describes the parts of the uncertainty map in more detail.

## Mitigating Uncertainty: Exploring the Use of the Guidance through a Real-World Case Study

There is no such thing as complete certainty when it comes to health care. Good quality research will help, but cannot entirely solve uncertainties in decision making for market authorization or resource allocation (33). Different root causes (visualized as the building blocks) of uncertainty in different domains (visualized in the uncertainty map) may call for different mitigation and management strategies and the stakeholders should agree what risks can be mitigated and in what way. This section will illustrate how the guidance may facilitate the nuanced discussion between stakeholders based on a recent case study.

### Case Study as Provided by a Health Technology Developer

The guidance was applied to a case study that comprised an oncology product intended as second line treatment for patients that had not responded to the prior first line treatment (standard of care (SoC)), in an indication with a generally recognized unmet medical need and an inadequate response to first line treatment. A full randomized clinical trial (RCT), comparing the SoC (Drug B) with the provisional second line treatment (Drug A) was being conducted. During the conduct of the pivotal trial, a new treatment (Drug C) entered the market and became the new SoC. Because the patients in the trial were primarily treated with the old SoC (Drug B), with few patients enrolled representing the new SoC (Drug C), the relevant regulatory authority proposed recommending the new therapy as the second line treatment only for patients that had previously received the old SoC (Drug B). The developer, therefore, faced a decision on how to reflect the SoC change to Drug C in the evidence base. The decision trade-off was balancing between a more “perfect” evidence base and the requirement of increased resources, timely patient access, and the risk of a further evolving treatment landscape. The options were to conduct a full new RCT, do a noncomparative single-arm trial with patients that had progressed on the new first line SoC (Drug C), or continue as planned with the RCT covering the old SoC as the comparator. The developer chose to continue the RCT with Drug B, and in addition, conduct a noncomparative trial in patients pretreated with Drug C to address the arisen uncertainties but avoid delaying patient access. The strategy resulted in some HTA organizations perceiving uncertainty due to the lack of a robust data package reflecting the Drug C as SoC, which would have required starting a new full RCT. On the other hand, other HTA organizations considered that there was no reason to believe the extrapolated efficacy of the second line treatment would not be accurate, due to a similar mode of action targeting the same gene of the two first line SoCs.

**Figure 3.**
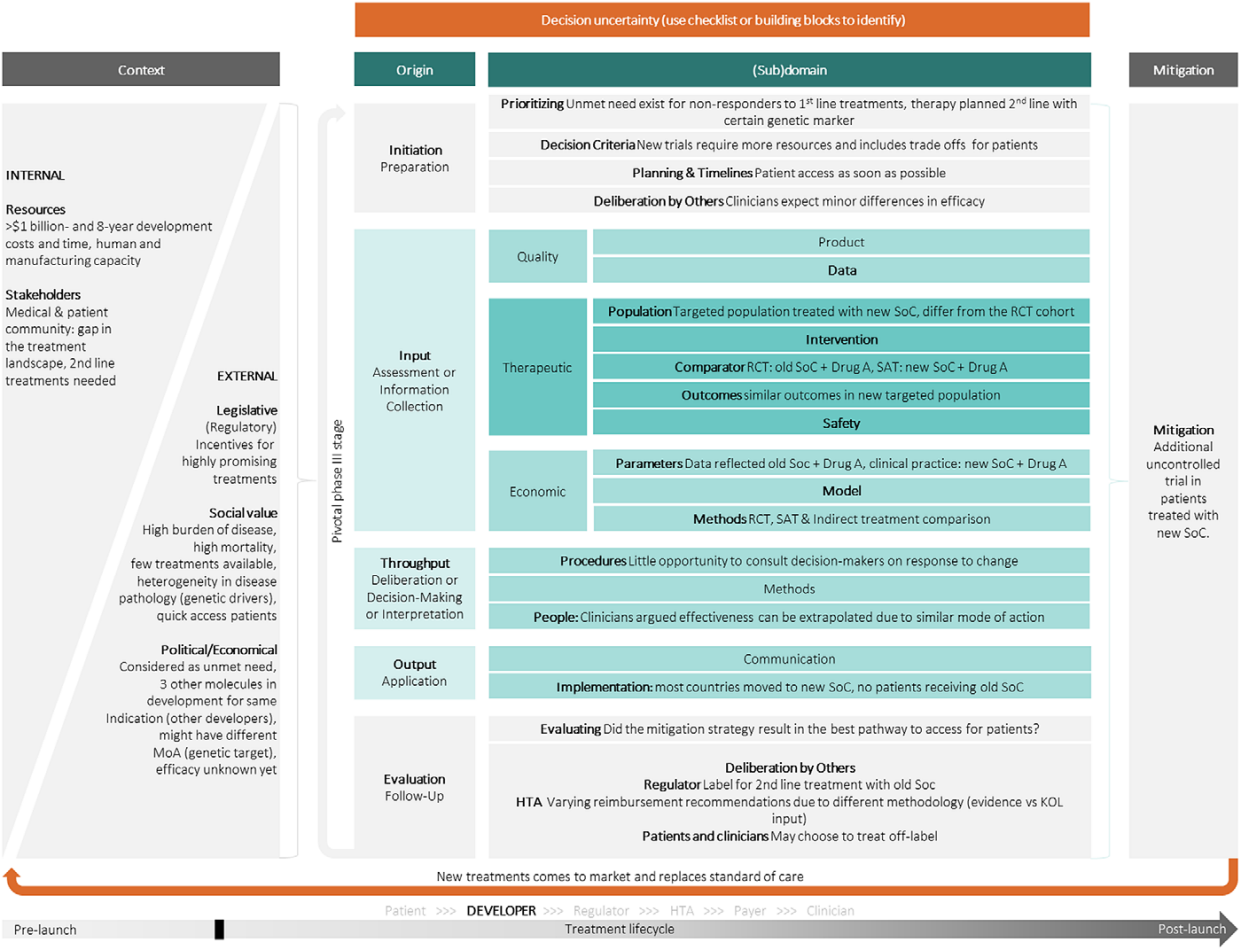
Populated uncertainty map based on a real-world case study by one of the participants.

In conclusion, the SoC change triggered uncertainties that influenced both regulatory and HTA decision making with an impact for the developer, patients, clinicians, and payers. We, therefore, use this example to demonstrate how the uncertainty guidance functions and how it could foster discussion around uncertainties. The completed map in Figure 3 demonstrates the uncertainty assessment from the industry perspective at time of the pivotal trial.

Identifying uncertainties with the proposed uncertainty map was considered to facilitate deciding on strategies to manage or mitigate them. To assess whether a mitigation or management strategy is needed, the authors recommend using the checklist (Table 2) to assess whether (i) the perceived uncertainty is relevant for decision making, (ii) it may lead to a decision error, and (iii) what the impact of that decision error is.

Reporting on uncertainties by means of the map may provide learnings for similar events and how they can be mitigated through new processes or tools. In the presented case, the developer could have anticipated some of the potential changes leading to perceived uncertainty. Equally for other stakeholders, the uncertainties may be slightly different than those described from the industry perspective. However, there are currently relatively few mechanisms to manage the anticipated change operationally, and the opportunity to have a more frequent discussion about mitigation is also lacking. There is a range of different mitigation or management strategies that could be applied in such a situation (e.g., post-launch evidence collection through a registry study, clinical trial, or managed entry agreement) but all of the choices made will have trade-offs, not least from the patient perspective, and without an opportunity for a dialogue between the stakeholders, it is difficult to choose the best strategies.

A dedicated discussion on mitigation and management strategies will be subject to a separate guidance from the working group.

## Discussion

It has been critical, and it will become increasingly more so, to develop approaches for multistakeholder discussion in the field of regulatory and HTA-informed decision making. New interventions are increasingly either complex, personalized, or constitute combination therapies and/or require companion diagnostics (34;35). New types of data and methods to gather data for these interventions also bring new uncertainties (36). These uncertainties have already led to tailored approaches within regulatory and HTA decision making, such as different forms of conditional approval and conditional reimbursement mechanisms (10;35–38).

This paper provides guidance on key building blocks that were collectively perceived to define uncertainty. The building blocks are the theoretical foundation for a checklist that enables defining whether any issue constitutes a relevant uncertainty. A regulatory-HTA relevant taxonomy, including domains in which uncertainty may exist, was developed to facilitate the categorization of uncertainties. The checklist and uncertainty map can be used by checking for each of the domains in the map whether there are relevant uncertainties with risks worth mitigating, to foster deliberation on the mitigation strategies for these uncertainties.

An important note on this guidance is that it starts with a theoretical exercise to define uncertainty. The resulting building blocks distinguish between nonpreventable (random variation) and theoretically preventable uncertainties (unavailable, inaccurate, conflicting, not understandable) (22;39). Uncertainties may be theoretically preventable; this is not always the case in practice. The preventable nature in practice is dependent on the contextual factors surrounding the decision at hand. An obvious example may be the theoretical possibility to increase patient numbers to improve the power of trial results. Practically this could not be feasible if the patients are simply not there. More implicitly, it may theoretically be possible to perform longer trials to include all possible outcome measures or to assess full trial populations, but in practice, there might be outweighing reasons not to do this (e.g., ethical considerations, financial resources, opportunity costs). This guidance specifically aims to facilitate deliberation about the nuances and the weighing of options for preventing, reducing, or mitigating uncertainty across stakeholders.

The paper distinguishes between uncertainties and risks. The highly context-specific nature of uncertainty may also impact the tolerance for risk that stakeholders may have. This may explain how one event causes different uncertainties to different stakeholders at different times, as well as how similar uncertainties are perceived and addressed differently by different decision makers, due to different remits. The risk tolerance may, for example, also be higher when it concerns children or disadvantaged populations or in case of urgent health-related societal needs. Again, such considerations may impact the resulting desired options for uncertainty mitigation, indicating the complexity of identifying, categorizing, and mitigating uncertainty in a multistakeholder context (40).

This paper focuses on the interface between regulatory and HTA-informed decision-making processes for drugs. We believe that this guidance could be applied in other decision-making processes such as the combination of diagnostics and drugs and/or diagnostics and medical devices or medical devices only, as elements similar to the theoretical building blocks of uncertainty listed in this paper are widely found in other scientific fields (41–43). However, this needs to be further investigated. Additionally, future research should focus on systematically mapping the available mitigation strategies and developing a method to link mitigation strategies to uncertainties, whether based on the building blocks of uncertainty or the domains of uncertainty.

## Conclusion

The systematic approach for identification of uncertainties and the checklist provided in this guidance has the potential to facilitate the understanding of uncertainty and its management by different stakeholders related to drugs in the regulatory-HTA interface. This can improve consistency and transparency throughout decision-making processes, and foster more alignment of approach (though not necessarily decision outcomes) between regulatory and HTA perspectives. To further support uncertainty management, linkage to suitable mitigation strategies and multistakeholder collaboration to develop them, is necessary.

## Supplementary material

For supplementary material accompanying this paper visit https://doi.org/10.1017/S0266462323000375. click here to view supplementary material
