# Supplementary material for: Uncertainty management in regulatory and health technology assessment decision-making on drugs: guidance of the HTAi-DIA Working Group
Source: Int J Technol Assess Health Care. 2023 Jun 16;39(1):e40. doi: 10.1017/S0266462323000375 (PMC11570246; doi:10.1017/S0266462323000375)
Supplement: Supplementary file 1 [file S0266462323000375sup.zip › S0266462323000375sup001.docx]

**Supplementary Table 1. Case studies survey.**

Potential uncertainties from each case study:

| **Case study** | **Uncertainty extracted from assessment reports (modified)** |
| --- | --- |
| Tumor agnostic therapy | “There are many small sub-populations, all with a very different prognosis based on each tumor location.” |
|  | “The natural history of disease for patients with a NTRK gene fusion is unknown.” |
|  | “The decision was informed by solely interim data.” |
|  | “A basket trial design was used without a comparator arm, an intra-person outcome measure was used as analysis control.” |
|  | “It is unclear which proportion of eligible patients will receive diagnostic testing in clinical practice.” |
| Treatment for cystic fibrosis | “While there was a statistically significant improvement for the primary endpoint, it was modest. Given the modest effect size, it is uncertain if it represents a clinically meaningful benefit above placebo.” |
|  | “While the numbers are small, the serious adverse events and discontinuation data suggest that the treatment’s use may be associated with liver toxicity.” |
|  | “The use in pregnant or lactating women has not been studied in adequate and well controlled trials.” |
|  | “It was noted that because the company’s trials were short, the long-term effects were uncertain.” |
|  | “It was stated that because both trials included people with mild to moderate cystic fibrosis, the clinical evidence may not be generalizable to people with severe cystic fibrosis or people with very mild cystic fibrosis.” |
|  | “Contradicting reimbursement recommendations in countries lead to confusion among clinicians and patients.” |

Survey questions for each of the potential uncertainties:

1. Do you consider this an ‘uncertainty’ (rather than an issue, a choice or something else)?
   1. Yes/No
2. Please briefly explain why.
   1. Open
3. In which domain would you classify this uncertainty/choice (e.g., input, throughput, output, population, intervention, comparator, outcome, study design, decision, deliberation, etc.)? Providing alternative/multiple options is allowed.
   1. Open
4. As which type would you classify this uncertainty/choice (e.g., parametric, structural, methodological, natural variability, heterogeneity, value, etc.)? Providing alternative/multiple options is allowed.
   1. Open
