# Supplementary material for: Uncertainty management in regulatory and health technology assessment decision-making on drugs: guidance of the HTAi-DIA Working Group
Source: Int J Technol Assess Health Care. 2023 Jun 16;39(1):e40. doi: 10.1017/S0266462323000375 (PMC11570246; doi:10.1017/S0266462323000375)
Supplement: Supplementary file 1 [file S0266462323000375sup.zip › S0266462323000375sup002.docx]

**Supplementary Table 2.** **Definitions used for the building blocks.**

| **Uncertainty can be defined as…** | **Number of votes** |
| --- | --- |
| A lack of sufficiently accurate information.* | 6 |
| The range of possible values within which the true value of the measurement lies.* | 2 |
| A deficiency in the information available for a given question, such that the conclusion, decision or recommendation is unknown or not definite | 1 |
| A lack of relevant knowledge about a particular outcome | 1 |
| Uncertainty describes limitations in knowledge that reduce decision maker confidence in predicting the impacts of a specific decision.* | 1 |
| Uncertainty means the "risk" that a decision based on the available evidence is wrong | 1 |
| Unpredictability of what the results could be on the basis of existing knowledge | 1 |
| A lack of precise knowledge.* | 1 |
| A situation in which something is not known, or something that is not known or certain. |  |
| Not knowing what to do or believe, or not able to decide about something, not known or fixed, not completely certain. |  |
| A feeling that blocks or delays decisions |  |
| The inability to act deterministically owing to lack of cause-effect understanding |  |
| A situation in which one has no knowledge about which of the several states of nature has occurred or will occur |  |
| The difference in the amount of information required to perform a task and the amount of information already possessed by the organization |  |
| A general term referring to all types of limitations in available knowledge that affect the range and probability of possible answers to an assessment question |  |
| Lack of precise knowledge about the likelihood of events |  |
| Accuracy: In the context of a study, the quality of a measurement (e.g., the mean estimate of a treatment effect) that is correct or that reflects the actual effectiveness of the treatment. Note: Not to be confused with preciseness: an estimate can be accurate, yet not be precise, if it is based on an unbiased method that shows great random variations (severity of the disease, coexisting conditions). |  |
| A measure of the likelihood of random errors in the results of a study, meta-analysis or measurement. The less random error the greater the precision. Confidence intervals around the estimate of effect from each study are one way of expressing precision, with a narrower confidence interval meaning more precision.  GRADE’s adopted definition of *certainty* of the evidence. Note that ‘‘quality of  evidence’’ refers to the same concept as ‘‘certainty of evidence’’: In the context of a systematic review, the ratings of the quality of evidence reflect the extent of our confidence that the estimates of the effect are correct. In the context of making recommendations, the quality ratings reflect the extent of our confidence that the estimates of an effect are adequate to support a particular decision or recommendation. |  |
| *Certainty* of evidence is best considered as the certainty that a true effect lies on one side of a specified threshold or within a chosen range. We define possible approaches for choosing threshold or range. |  |
| Ignorance (not knowing what will occur), uncertainty (knowing what will occur without knowledge of the probabilities) and some degree of certainty (knowing what will occur and the probability of its occurrence) |  |
| Eliciting measures of uncertainty can be complicated, particularly as one wants to ensure that quantities reflect uncertainty in the expected value rather than variability or heterogeneity. |  |

* Definition suggested by a working group member.
