# Supplementary material for: Uncertainty management in regulatory and health technology assessment decision-making on drugs: guidance of the HTAi-DIA Working Group
Source: Int J Technol Assess Health Care. 2023 Jun 16;39(1):e40. doi: 10.1017/S0266462323000375 (PMC11570246; doi:10.1017/S0266462323000375)
Supplement: Supplementary file 1 [file S0266462323000375sup.zip › S0266462323000375sup003.docx]

**Supplementary Table 3. Alphabetic list of articles selected through the scoping review.**

| Afzali HHA, Karnon J. Exploring structural uncertainty in model-based economic evaluations. Pharmacoeconomics. 2015 May;33(5):435–43. |
| --- |
| Annemans L, Makady A. TRUST4RD: tool for reducing uncertainties in the evidence generation for specialised treatments for rare diseases. Orphanet J Rare Dis. 2020 May 26;15(1):127. |
| Bloem LT, Vreman R, Peeters NWL, Hoekman J, Elst ME van der, Leufkens H, et al. Associations between uncertainties identified by the European Medicines Agency and national decision making on reimbursement by HTA agencies. 2021; Available from: https://www.semanticscholar.org/paper/745602fb2d8b7c3c2389cbd91cd9bf854155f129 |
| Bojke L, Grigore B, Jankovic D, Peters J, Soares M, Stein K. Informing Reimbursement Decisions Using Cost-Effectiveness Modelling: A Guide to the Process of Generating Elicited Priors to Capture Model Uncertainties. Pharmacoeconomics. 2017 Sep;35(9):867–77. |
| Bond K, Stiffell R, Ollendorf DA. Principles for deliberative processes in health technology assessment. Int J Technol Assess Health Care. 2020 Aug 4;1–8. |
| Bouvy J, Sapède C, Garner S. Managed Entry Agreements for Pharmaceuticals in the Context of Adaptive Pathways in Europe. 2018; Available from: https://www.semanticscholar.org/paper/cf47b7732a0f6fa0d13858f4f726b8b9d6233e73 |
| Brown P, Calnan M. NICE technology appraisals: working with multiple levels of uncertainty and the potential for bias. Med Health Care Philos. 2013 May;16(2):281–93. |
| Chalmers I. Confronting therapeutic ignorance. BMJ. 2008 Jul 16;337:a841. |
| Collins R, Bowman L, Landray M, Peto R. The Magic of Randomization versus the Myth of Real-World Evidence. N Engl J Med. 2020 Feb 13;382(7):674–8. |
| Coory M. TIDI addresses uncertainty but not indeterminism. Value Health. 2013 Feb;16(1):215–6. |
| Dhruva S, Darrow JJ, Kesselheim A, Redberg R. Strategies to Manage Drugs and Devices Approved Based on Limited Evidence: Results of a Modified Delphi Panel. 2022; Available from: https://www.semanticscholar.org/paper/6d7d415f556cdef977bf75977b2a1142d35ebb12 |
| Dobrow MJ, Goel V, Lemieux-Charles L, Black NA. The impact of context on evidence utilization: A framework for expert groups developing health policy recommendations. Social Science & Medicine. 2006 Oct 1;63(7):1811–24. |
| Drummond M. When do performance-based risk-sharing arrangements make sense? Eur J Health Econ. 2015 Jul;16(6):569–71. |
| Eichler H, Bloechl-Daum B, Brasseur D, Breckenridge A, Leufkens H, Raine J, et al. The risks of risk aversion in drug regulation. 2013; Available from: https://www.semanticscholar.org/paper/f8499d79dc8f04e2521911e405a7ede621f047c4 |
| Eichler H, Pignatti F, Flamion B, Leufkens H, Breckenridge A. Balancing early market access to new drugs with the need for benefit/risk data: a mounting dilemma. 2008; Available from: https://www.semanticscholar.org/paper/0ef1a9e5c1acc38ce41d0da3e47689b59f0acbd4 |
| Eichler HG, Pignatti F, Schwarzer-Daum B, Hidalgo-Simon A, Eichler I, Arlett P, et al. Randomized Controlled Trials Versus Real World Evidence: Neither Magic Nor Myth. Clin Pharmacol Ther. 2021 May;109(5):1212–8. |
| Facey K, Whittal A, Drummond M, Upadhyaya S, Junghans T, Nicod E. HTA Appraisal Framework Suitable for Rare Disease Treatments [Internet]. England: Impact HTA; 2021 May. (WP10 Guidance to support consistent HTA appraisal for orphan medicinal products (OMPs)). Report No.: 10.1. Available from: https://www.impact-hta.eu/project-outputs |
| Farmer A, Milne R, Walley T. Research to decrease areas of clinical uncertainty. BMJ. 2011 Feb 4;342:d369. |
| Ferrario A, Kanavos P. Dealing with uncertainty and high prices of new medicines: a comparative analysis of the use of managed entry agreements in Belgium, England, the Netherlands and Sweden. 2015; Available from: https://www.semanticscholar.org/paper/32ebcfa85cbdbbdbddca23e67be0c461c05d57a8 |
| Ghabri S, Cleemput I, Josselin J. Towards a New Framework for Addressing Structural Uncertainty in Health Technology Assessment Guidelines. 2018; Available from: https://www.semanticscholar.org/paper/a3039a4f6fc3de2d32e3156f903d35f90cfd3778 |
| Ghabri S, Hamers FF, Josselin JM. Exploring Uncertainty in Economic Evaluations of Drugs and Medical Devices: Lessons from the First Review of Manufacturers’ Submissions to the French National Authority for Health. Pharmacoeconomics. 2016 Jun;34(6):617–24. |
| Goeree R, Levin L, Chandra K, Bowen JM, Blackhouse G, Tarride JE, et al. Health technology assessment and primary data collection for reducing uncertainty in decision making. J Am Coll Radiol. 2009 May;6(5):332–42. |
| Grimm S, Pouwels X, Ramaekers B, Wijnen B, Knies S, Grutters J, et al. Development and Validation of the TRansparent Uncertainty ASsessmenT (TRUST) Tool for Assessing Uncertainties in Health Economic Decision Models. 2019; Available from: https://www.semanticscholar.org/paper/bbd2c0b15fffa12c2095345aa19e00884e73b8e4 |
| Grimm S, Pouwels X, Ramaekers B, Wijnen B, Knies S, Grutters J, et al. Building a trusted framework for uncertainty assessment in rare diseases: suggestions for improvement (Response to “TRUST4RD: tool for reducing uncertainties in the evidence generation for specialised treatments for rare diseases”). 2021; Available from: https://www.semanticscholar.org/paper/71837ac8452e0e904cac73f38741840ad74f0faa |
| Grimm SE, Pouwels X, Ramaekers BLT, Wijnen B, Otten T, Grutters J, et al. State of the ART? Two New Tools for Risk Communication in Health Technology Assessments. Pharmacoeconomics. 2021 Oct;39(10):1185–96. |
| Grutters J, Asselt MV, Chalkidou K, Joore M. Healthy Decisions: Towards Uncertainty Tolerance in Healthcare Policy. 2014; Available from: https://www.semanticscholar.org/paper/1e6b3c03cc837dfc9bce1e898c86677f039d0b96 |
| Gutiérrez-Ibarluzea I, Chiumente M, Dauben HP. The Life Cycle of Health Technologies. Challenges and Ways Forward. Front Pharmacol. 2017 Jan 24;8:14. |
| Halldorsson T, Jeger MJ, Knutsen HK, More S, Naegeli H, Noteborn H, et al. Guidance on Uncertainty Analysis in Scientific Assessments. EFSA Journal. 2018;16(1):e05123. |
| Hulstaert F, Pouppez C, Primus-de Jong C, Harkin K, Neyt M. Evidence gaps for drugs and medical devices at market entry in Europe and potential solutions [Internet]. Brussels: Belgian Health Care Knowledge Centre (KCE); 2021 [cited 2022 Aug 3] p. KCE Reports 347. Report No.: KCE Reports 347. Available from: https://kce.fgov.be/en/evidence-gaps-for-drugs-and-medical-devices-at-market-entry-in-europe-and-potential-solutions |
| Hultcrantz M, Rind D, Akl EA, Treweek S, Mustafa RA, Iorio A, et al. The GRADE Working Group clarifies the construct of certainty of evidence. J Clin Epidemiol. 2017 Jul;87:4–13. |
| Janiaud P, Irony T, Russek-Cohen E, Goodman SN. U.S. Food and Drug Administration Reasoning in Approval Decisions When Efficacy Evidence Is Borderline, 2013-2018. Ann Intern Med. 2021 Nov;174(11):1603–11. |
| Jansen E, Hines PA, Berntgen M, Brand A. Strengthening the Interface of Evidence-Based Decision Making Across European Regulators and Health Technology Assessment Bodies. 2022; Available from: https://www.semanticscholar.org/paper/dcf68894678134286538fe66f51f1b48929fd782 |
| Janssens R, Zafiropoulos N, Koenig F, Kouroumalis A, Guizzaro L, Pignatti F, et al. HOW DO REGULATORS MANAGE UNCERTAINTY? A CLASSIFICATION OF UNCERTAINTIES AND COPING STRATEGIES. 2020. |
| Kanavos P, Ferrario A, Tafuri G, Siviero P. Managing Risk and Uncertainty in Health Technology Introduction: The Role of Managed Entry Agreements. 2017; Available from: https://www.semanticscholar.org/paper/88e45b10f0cc7a0c06a729fab577ffc7ed0a1c24 |
| Kirwin E, Round J, Bond K, McCabe C. A Conceptual Framework for Life-Cycle Health Technology Assessment. Value in Health. 2022 Jul 1;25(7):1116–23. |
| Kotecha D, Asselbergs FW, Achenbach S, Anker SD, Atar D, Baigent C, et al. CODE-EHR best practice framework for the use of structured electronic healthcare records in clinical research. BMJ. 2022 Aug 29;378:e069048. |
| Lynch H, Robertson CT. Challenges in confirming drug effectiveness after early approval. 2021; Available from: https://www.semanticscholar.org/paper/229ce0c0e0a50e36f26de732bf5ce9fb0005dc34 |
| Maynou L, Cairns J. An empirical analysis of Drug Reimbursement Decisions in 6 European countries *. 2017; Available from: https://www.semanticscholar.org/paper/e230983435651a930d1f537fe909030ba9236209 |
| Maynou L, Cairns J. What is driving HTA decision-making? Evidence from cancer drug reimbursement decisions from 6 European countries. 2019; Available from: https://www.semanticscholar.org/paper/6a4825ed7f38ec91ee03d0edad486e96e8a983fb |
| Merész G, Dóczy V, Hölgyesi Á, Németh G. A critical assessment framework to identify, quantify and interpret the sources of uncertainty in cost-effectiveness analyses. BMC Health Serv Res. 2022 Jun 25;22(1):822. |
| National Institute for Health and Care Excellence. Guide to the Methods of Technology Appraisal 2013 [Internet]. London: National Institute for Health and Care Excellence (NICE); 2013 [cited 2022 Aug 3]. (NICE Process and Methods Guides). Available from: http://www.ncbi.nlm.nih.gov/books/NBK395867/ |
| Nicod E, Maynou L, Visintin E, Cairns J. Why do health technology assessment drug reimbursement recommendations differ between countries? A parallel convergent mixed methods study. 2019; Available from: https://www.semanticscholar.org/paper/105500818bf6abe0f4e1edd7599bf877fb7384d2 |
| Nicod E, Berg Brigham K, Durand-Zaleski I, Kanavos P. Dealing with Uncertainty and Accounting for Social Value Judgments in Assessments of Orphan Drugs: Evidence from Four European Countries. Value Health. 2017 Aug;20(7):919–26. |
| Oortwijn W, Jansen M, Baltussen R. Use of Evidence-Informed Deliberative Processes by Health Technology Assessment Agencies Around the Globe. Int J Health Policy Manag. 2020 Jan 1;9(1):27–33. |
| Petersohn S, Grimm S, Ramaekers B, Cate‐Hoek AT ten, Joore M. Exploring the Feasibility of Comprehensive Uncertainty Assessment in Health Economic Modeling: A Case Study. 2021; Available from: https://www.semanticscholar.org/paper/152705ecf8dd133e6325654256cbc5d9c6d6939d |
| Pignatti F, Wilking U, Postmus D, Wilking N, Delgado J, Bergh J. The value of anticancer drugs - a regulatory view. Nat Rev Clin Oncol. 2022 Mar;19(3):207–15. |
| Pouwels X, Grutters J, Bindels J, Ramaekers B, Joore M. Uncertainty and Coverage With Evidence Development: Does Practice Meet Theory? 2019; Available from: https://www.semanticscholar.org/paper/9e5bf76310e7cc7ab02c7d056442d33b15c275f1 |
| Pujolràs LM, Cairns J. Why do some countries approve a cancer drug and others don’t? 2015; Available from: https://www.semanticscholar.org/paper/f56abcb63e85e57d1188bafbd17bb6daf5341565 |
| Putzeist M, Mantel‐Teeuwisse A, Aronsson B, Rowland M, Wied CG, Vamvakas S, et al. Factors influencing non-approval of new drugs in Europe. 2012; Available from: https://www.semanticscholar.org/paper/b92ee746708cba26b6d4d83c888649dd986614cd |
| Rothery C, Claxton K, Palmer S, Epstein D, Tarricone R, Sculpher M. Characterising Uncertainty in the Assessment of Medical Devices and Determining Future Research Needs. Health Econ. 2017 Feb;26 Suppl 1:109–23. |
| Salcher-Konrad M, Naci H, Davis C. Approval of Cancer Drugs With Uncertain Therapeutic Value: A Comparison of Regulatory Decisions in Europe and the United States. 2020; Available from: https://www.semanticscholar.org/paper/08648551ee7f9b8951ea0eb087b60111f3261a79 |
| Schünemann HJ, Reinap M, Piggott T, Laidmäe E, Köhler K, Pōld M, et al. The ecosystem of health decision making: from fragmentation to synergy. The Lancet Public Health. 2022 Apr 1;7(4):e378–90. |
| Stern AD. Innovation under Regulatory Uncertainty: Evidence from Medical Technology. J Public Econ. 2017 Jan;145:181–200. |
| Tervonen T, Angelis A, Hockley K, Pignatti F, Phillips LD. Quantifying Preferences in Drug Benefit-Risk Decisions. Clin Pharmacol Ther. 2019 Nov;106(5):955–9. |
| Trapani D, Tay-Teo K, Tesch ME, Roitberg F, Sengar M, Altuna SC, et al. Implications of Oncology Trial Design and Uncertainties in Efficacy-Safety Data on Health Technology Assessments. Curr Oncol. 2022 Aug 16;29(8):5774–91. |
| Trowman R, Powers A, Ollendorf DA. Considering and communicating uncertainty in health technology assessment. Int J Technol Assess Health Care. 2021 Jul 15;37(1):e74. |
| Vreman R, Mantel‐Teeuwisse A, Hövels A, Leufkens H, Goettsch W. Differences in Health Technology Assessment Recommendations Among European Jurisdictions: The Role of Practice Variations. 2020; Available from: https://www.semanticscholar.org/paper/ea2193438c153ed1d7c1b61090c573944b86b73e |
| Vreman R, Naci H, Goettsch W, Mantel‐Teeuwisse A, Schneeweiss S, Leufkens H, et al. Decision Making Under Uncertainty: Comparing Regulatory and Health Technology Assessment Reviews of Medicines in the United States and Europe. 2020; Available from: https://www.semanticscholar.org/paper/645b58b95e2bd8da0841fc8311b38631cc89312a |
| Vreman RA, Bouvy JC, Bloem LT, Hövels AM, Mantel-Teeuwisse AK, Leufkens HGM, et al. Weighing of Evidence by Health Technology Assessment Bodies: Retrospective Study of Reimbursement Recommendations for Conditionally Approved Drugs. Clin Pharmacol Ther. 2019 Mar;105(3):684–91. |
| Vreman RA, Strigkos G, Leufkens HGM, Schünemann HJ, Mantel-Teeuwisse AK, Goettsch WG. Addressing uncertainty in relative effectiveness assessments by HTA organizations. Int J Technol Assess Health Care. 2022 Jan 31;38(1):e17. |
| Walker S, McAuslane N, Liberti L, Leong J, Salek S. A Universal Framework for the Benefit-Risk Assessment of Medicines. 2015; Available from: https://www.semanticscholar.org/paper/95aa7b7aa00dee30300cacfbfd332ed350cffbbf |
| Walker S, Sculpher M, Claxton K, Palmer S. Coverage with evidence development, only in research, risk sharing, or patient access scheme? A framework for coverage decisions. Value Health. 2012 May;15(3):570–9. |
| Wallach J, Ross J, Naci H. The US Food and Drug Administration’s expedited approval programs: Evidentiary standards, regulatory trade-offs, and potential improvements. 2018; Available from: https://www.semanticscholar.org/paper/eeb27befd66abc3e064503b0e3f3ea0de3a935c5 |
| Woodcock J. Evidence vs. Access: Can Twenty‐First‐Century Drug Regulation Refine the Tradeoffs? 2012; Available from: https://www.semanticscholar.org/paper/6dcf1b38db5145796d088229903e258659de1145 |
| Wranik WD, Gambold L, Peacock S. Uncertainty tolerance among experts involved in drug reimbursement recommendations: Qualitative evidence from HTA committees in Canada and Poland. Health Policy. 2021 Mar;125(3):307–19. |
| HTA Core Model® Online \| Home [Internet]. [cited 2022 Oct 26]. Available from: http://corehta.info/ |
