# Supplementary material for: Uncertainty management in regulatory and health technology assessment decision-making on drugs: guidance of the HTAi-DIA Working Group
Source: Int J Technol Assess Health Care. 2023 Jun 16;39(1):e40. doi: 10.1017/S0266462323000375 (PMC11570246; doi:10.1017/S0266462323000375)
Supplement: Supplementary file 1 [file S0266462323000375sup.zip › S0266462323000375sup004.docx]

**Supplementary Table 4. Detailed explanation of all the domains in the uncertainty map.**

| Context (internal) | |  |
| --- | --- | --- |
|  | Risk aversion | Risk aversion affects mitigation trade-offs and is dependent on individual’s preferences as well as the decision-question, for example the population or the disease assessed. In pediatric indications decision-makers may be more risk averse whereas in later stage palliative treatments they may be more risk accepting, leading to different choices for mitigation strategies. |
|  | Governance | Governance determines how decision-making body is to be governed and through which processes a given decision is to be made. In other words, it determines both structure (which committees, boards, advisory groups, the composition of such committees, etc.) and processes (how such bodies are to be formed, how they will (be) convened and under which terms of reference they will decide), or it can relate to leadership and organizational culture. |
|  | Social value | Social value relates to the values underlying the health systems that are related to affordability, quality and accessibility based on medical necessity, cultural values and societal norms. These could include arguments about necessity (e.g., disease severity, unmet medical need, dignity, and human rights), as well as the societal expectations and desires towards new treatments and decision-making institutions. May also include media reporting on health care/treatments, it includes cultural values of society that may differ among small/large, rich/low-income countries or other (religious regions). Societal norms for public and private discourse. |
|  | Resources | Resources may include personnel, financial resources, time, knowledge and skills of appropriate methods or other tools. |
|  | Ethics | Ethical considerations may include equality of access to the best possible healthcare provision between different population groups and/or in different disease areas, affordability or it may affect trial design by in-or exclusion of patients, chosen comparative treatments or duration of the trials. |
|  | Trust | Trust among stakeholders is important, as it may affect the trust in the quality of the information received from stakeholders and therefore induce uncertainty or affect uncertainty mitigation. For example, this may relate to the trust in (the correct use of) new complex statistical methods. |
| Context (external) | |  |
|  | Political | The political context in democratic societies aims to have legitimate decision-making and this would necessitates transparent and informed stakeholder dialogue through deliberative mechanisms that safeguard representativeness. |
|  | Economic | Economic context may mostly affect the resources available for decision-making or the availability and affordability of new drugs. |
|  | Legislative | The legislation that may be in place, defining for example which procedures should be followed, how much time assessments may take, constitutional right, legal mandates etc. |
|  | Social value | Social value affects both internal as well as external factors as the social values of internal assessors or decision or policy makers may have an influence on the perception of uncertainty as well as social value among society, outside the organization. This should encompass also the distinction between certain societal values as defined in the law and implicit or personal societal values. |
|  | Stakeholders | Communication from and to stakeholders, and the kind of information that is received from up-stream parties and what is passed on to down-stream decision-makers affects uncertainty as it may influence the comprehension of uncertainty and is inextricably linked to trust. It also includes the availability of stakeholders. This could also be awareness of what other stakeholders are doing exactly, why they arrive at certain decisions or recommendations. Additionally, uncertainties affected by stakeholders may be due to expectations from the other stakeholders, regarding decisions, submissions, or communication. |
|  | Organizational | Organizational aspects relate to ‘soft’ rules such as the existence of mechanisms to use evidence in decision making (mandates), organization of the healthcare system (centralized or decentralized elements). |
| Initiation | |  |
|  | Prioritizing | The initiation phase relates to the uncertainties in the decisions that were made before the situation in which the uncertainty map is used (e.g., the exact definition of the population or indication), that will affect the input, throughput or output. These relate to the contextual factors, though are distinguished as these can be influenced, whereas contextual factors cannot. Examples could be the prioritization of products for certain regulatory pathways, a full HTA assessment including pharmaco-economic analysis or criteria for products that qualify for an orphan designation. |
|  | Decision Criteria | Decision criteria, meaning methods to assess and criteria that products must fulfil for market authorization or reimbursement, are usually developed before submission. For example the decision to use GRADE methodology to assess data quality or to make use of scientific advice to get input on trial design and other data generation. |
|  | Planning and timelines | The timelines, for example in an accelerated versus a normal pathway may affect the moment that patients have access but may also affect the thoroughness of assessments. |
|  | Deliberation by others | Lastly, the deliberation by others may affect the assessment process, for example a conditional market authorization resulting in earlier data cut-offs. Uncertainty may exist around the impact of all of these decisions. |
| Input (quality) | |  |
|  | Product | The quality aspect is two-fold. It may include the regulatory quality assessment of the product with topics as described in the European Public Assessment reports (regulatory focused). |
|  | Data | Additionally, it relates to the quality of the studies or other data underlying the decision. The trial design in terms of duration of follow-up, randomization, (double) blindness, basket designs or more pragmatic approaches or decentralized data generation would fall in this category. |
| Input (therapeutic) | |  |
|  | Population | The key part of the uncertainty map is the therapeutic area, including all the PICO elements; the patient population, the intervention, the comparator, outcomes and separately mentioned the safety outcomes as this is such an important part of the assessment in the regulatory decisions. Under population would fall the characteristics of the patients with the assessed indication, including natural history, earlier treatments, age, sex, the relation between the patients included in trials versus the population treated in practice. |
|  | Intervention | The intervention also includes the diagnostics before a treatment can be given, the dosage, treatment duration or stop criteria. |
|  | Comparator | The comparator relates to the standard of care and how the trial comparator matches with that. |
|  | Outcomes | Outcomes relate to all the hard, surrogate, and patient reported outcomes relevant to regulators, HTA, clinicians and patients. |
|  | Safety | Safety relates to any unwanted or harmful effects caused by using the drug. |
| Input (economic) | |  |
|  | Cost parameters | More specific to HTA , the third domain in input relates to the cost parameters, which may include all costs required in the pharmaco-economic models, direct and indirect and medical and non-medical costs (such as costs related to unemployment due to illness). Cost-effectiveness analysis may provide a perception of less uncertainty by putting uncertainty on a spectrum, rather than stating it as binary. |
|  | Model structure | As cost-effectiveness analyses uses models and inherent to models is that it is a simplification of reality, thus inherently introducing uncertainty related to the chosen model and how well it mimics reality. Of course, as more complex models require more detailed data sources that may not always be available, the complexity of the model does not automatically guarantee a better proxy of reality. |
|  | Methods | Finally, the methods used, such as the chosen discount rates or the perspective chosen, may affect uncertainty as they are. |
| Throughput | |  |
|  | Procedures | Institutional procedures and methods for deliberation(33). For example, methods for considering contextual factors, format and consistency of uncertainty explorations and visualization, presentation of information to be deliberated on, weighing trade-offs for mitigating uncertainty in deliberative processes. |
|  | Methods | It also includes considerations the level of uncertainty that is acceptable for participants of deliberative processes, as this is largely dependent on their appetite for risk. |
|  | People | The people deliberating also largely affect uncertainty as knowledge and skills may vary. |
| Output | |  |
|  | Communication | Communication of uncertainties in a concise and understandable way to other stakeholders. |
|  | Implementation | The implementation of new treatments in clinical guidelines, prescription behavior according to guidelines, uptake of new methods for assessment or deliberation. This may include contextual issues related to health care structures (e.g., [de]centralized care, connection between primary, secondary, or tertiary care, national versus private insurance, patient preferences and adherence to treatment). |
| Evaluation | |  |
|  | Evaluation | This may include the monitoring of the risks that were considered in the trade-off for mitigating uncertainty and deciding on whether to perform a re-assessment. It creates the opportunity to steer and reassess, although it may not always be clear in which situations a reassessment is really of added value, i.e., would an initial decision change based upon reassessment. In other words, checking whether all stakeholders can ‘live with’ the mitigation strategies that have been decided on, it is part of the life cycle approach. |
|  | Deliberation by others | As opposed to the deliberation by others in the initiation phase, referring to the use of previous deliberations in the current deliberation, this refers to the effect that the current deliberation may have on subsequent decision-makers. |
